# Apatinib Inhibits the Invasion and Metastasis of Liver Cancer Cells by Downregulating MMP-Related Proteins via Regulation of the NF-*κ*B Signaling Pathway

**DOI:** 10.1155/2020/3126182

**Published:** 2020-06-19

**Authors:** Xiaoxiao He, Zaozao Huang, Ping Liu, Qiuting Li, Mengmeng Wang, Mengjun Qiu, Zhifan Xiong, Shengli Yang

**Affiliations:** ^1^Division of Gastroenterology, Liyuan Hospital, Tongji Medical College, Huazhong University of Science and Technology, Wuhan 430077, China; ^2^Yangchunhu Community, Liyuan Hospital, Tongji Medical College, Huazhong University of Science and Technology, Wuhan 430077, China; ^3^Department of Orthopedics, Liyuan Hospital, Tongji Medical College, Huazhong University of Science and Technology, Wuhan 430077, China; ^4^Cancer Center, Union Hospital, Tongji Medical College, Huazhong University of Science and Technology, Wuhan 430022, China

## Abstract

**Objective:**

We aimed to investigate whether apatinib has an inhibitory effect on the invasion and metastasis of liver cancer in vitro.

**Methods:**

The anti-invasion and antimetastasis effects of apatinib in HepG2, Hep3B,Huh7 and SMMC-7721 liver cancer cell lines were tested by the wound-healing and transwell invasion assays. Real-time PCR and Western blot were used to detect the influence of apatinib on the gene expression of MMPs, TIMPs, and constituents of the NF-*κ*B signaling pathway in Hep3B and HepG2 liver cell lines.

**Results:**

Apatinib has a significant inhibitory effect on the metastasis and invasion of liver cancer cells. The expression levels of MMP-1, MMP-2, MMP-3, MMP-7, MMP-9, MMP-10, MMP-11, and MMP-16 were downregulated, while the expression levels of TIMP-3 and TIMP-4 were upregulated by apatinib treatment at both the mRNA and protein levels. The phosphorylation of I*κ*B*α* and NF-*κ*B p65 was significantly reduced compared with that in the control group.

**Conclusions:**

Apatinib inhibits the invasion and metastasis of human liver cancer cells by downregulating the expression of MMP-related genes. This may be achieved by inhibiting the activation of the NF-*κ*B signaling pathway.

## 1. Introduction

Hepatocellular carcinoma (HCC) is the most frequent primary cancer of the liver, which is the sixth most common cancer and the third leading cause of death in the world [1–3]. There is a main reason for the poor prognosis of patients with liver cancer. Liver cancer cells induce the expression of specific proteases, which cause the invasion and metastasis of tumor cells through degradation of the extracellular matrix (ECM) and basement membrane. Matrix metalloproteinases (MMPs) present the most important group of these proteases. As a large family of calcium-dependent, zinc-containing endopeptidases, MMPs play an important role in tumor invasion and metastasis [4–6]. Their activities are posttranslationally regulated by endogenous tissue inhibitors of metalloproteinases (TIMPs) [7, 8]. Therefore, the MMP/TIMP balance is critical for the inhibition of tumor invasion and metastasis.

Apatinib is a new type of small-molecule antiangiogenesis agent and a targeting tyrosine kinase inhibitor that selectively inhibits the vascular endothelial growth factor receptor-2 (VEGFR-2), thus suppressing tumor angiogenesis and being effective against tumors [9]. Currently, clinical studies have confirmed the effectivity and safety of apatinib in the treatment of advanced liver cancer [10, 11]. Furthermore, studies have shown that apatinib led to a survival benefit of patients with advanced gastric cancer [12]. The purpose of this study was to explore the effect of apatinib on metastasis, invasion, and expression of MMPs of liver cancer cells and to provide further theoretical basis for the clinical application of apatinib.

## 2. Materials and Methods

### 2.1. Reagents and Antibodies

Apatinib mesylate tablets (AITAN®; 425 mg/tablet) were purchased from Hengrui Medicine Company (Jiangsu, China). Tablets were dissolved in 100% dimethyl sulfoxide (DMSO; MP Biomedicals, Santa Ana, CA, USA) to a concentration of 4 mmol/L and stored at –20°C. The stock solution was diluted to a working concentration with DMEM (Gibco, Grand Island, NY, USA), and the final DMSO concentration in cell culture was less than 0.1%. The BCA kit was purchased from Google Biotechnology Ltd. (Wuhan, China). The RNA extraction reagent TRIzol and the PCR reagent Power SYBR® Green PCR Master Mix were purchased from Life Technologies (Grand Island, NY, USA), and the PCR primers were synthesized by Google Biotechnology Ltd. (Wuhan, China). Anti-MMP-1, anti-MMP-2, anti-MMP-3, anti-MMP-7, anti-MMP-9, anti-TIMP-1, anti-TIMP-2, and anti-TIMP4 antibodies were purchased from Proteintech Group (Chicago, USA), and anti-MMP-10, anti-MMP-11, and anti-MMP-16 antibodies were purchased from Biorbyt Ltd. (Cambridge, UK). Anti-TIMP-3 was purchased from Santa Cruz Biotechnology Inc. (Santa Cruz, CA, USA). An NF-*κ*B pathway sampler kit (9936T) was purchased from Cell Signaling Technology Inc. (Danvers, MA, USA).

### 2.2. Cells and Cell Culture

The HepG2, Hep3B, Huh7, and SMMC-7721 human liver cancer cell lines were purchased from the China Center for Type Culture Collection (Wuhan, China). Cells were cultured in DMEM cell culture medium containing 10% fetal bovine serum (FBS) and 1% penicillin-streptomycin. The medium was changed every three days, and the cells were passaged with a split ratio of 1 : 4 when they were 80–90% confluent.

### 2.3. Analysis of the Effect of Apatinib on Invasion and Metastasis of Human Liver Cancer Cells

#### 2.3.1. Wound-Healing Assay

HepG2, Hep3B, Huh7, and SMMC-7721 cells were homogeneously seeded in 6-well plates (2 × 10^5^ per well). After 24 h of growth, a monolayer of cells was attached to the bottom of the wells. The monolayer was gently scratched with a sterile 10 *μ*L pipette tip across the center of the well. The wells were washed three times to remove detached cells and replenished with fresh DMEM medium without serum. Cells were cultured in serum-free DMEM containing 20 or 40 *μ*mol/L apatinib for 48 h. Untreated cells were used as the blank control. Cells cultured only in serum-free DMEM for 48 h were used as the control group. Then, all cells were kept at 37°C in a humidified atmosphere containing 5% CO_2_. Optical microscopy was performed after 0, 24, and 48 h. The migration rate was calculated using the following formula: migration rate = (width at 0 h–width at 24 or 48 h)/width at 0 h. All experiments were performed in triplicate.

### 2.4. Transwell Invasion Assay

The invasion capacity of HepG2, Hep3B, Huh7, and SMMC-7721 cells was evaluated by the transwell invasion assay (Falcon, Oxford, UK) using a polycarbonate membrane filter with a pore size of 8.0 *μ*m. The transwell compartments were prepared by incubation with Matrigel (diluted to 1 : 100) at 37°C for 2 h. Then, the compartments were placed into 24-well plates. To the upper compartments, 1 × 10^5^ HepG2, Hep3B, Huh7, and SMMC-7721 cells were introduced, and 0, 20, or 40 *μ*mol/L apatinib in DMEM medium was added up to a total volume of 200 *μ*L. To the lower compartments, 600 *μ*L DMEM containing 10% FBS was added. All experiments were performed in triplicate. After incubation for 24 h, the transwell compartments were taken out and washed with PBS. Cells were fixed by polyformaldehyde at room temperature for 20 min, followed by crystal violet staining for 5 min and washing with deionized distilled water (ddH_2_O). Cells on the lower side of the filter were counted under a microscope, and the average counting from five random views was determined.

### 2.5. Quantitative Real-Time Reverse Transcription-PCR (qRT-PCR)

The effect of apatinib on the mRNA expression levels of MMP and TIMP genes was analyzed using real-time PCR. The cells were seeded in 6-well plates at a concentration of 10^5^ cells/mL and cultured for 24 h. Then, cells were exposed to 0, 20, or 40 *μ*mol/L apatinib for 48 h. The total RNA of the cells was extracted using the RNA extraction reagent TRIzol, and concentration and purity were determined using an ultraviolet spectrophotometer. The same amount of mRNA was used as a reverse transcriptional template to obtain the cDNA. Primers and SYBR Green Mix (2x) were used for quantitative PCR, and each reaction was performed in triplicate.

Fluorescence quantitative PCR was carried out in 20 *μ*L of a mixture containing 1.5 *μ*L cDNA template, 1 *μ*L of each primer (10 *μ*mol/L), 10 *μ*L SYBR Green Mix, and 10 *μ*L ddH_2_O. PCR for each sample was performed in triplicate. The following thermal cycling conditions were applied: initial denaturation at 94°C for 5 min, followed by 33 PCR amplification cycles at 94, 58, and 72°C for 1 min at each temperature, and termination by an extension step at 72°C for 10 min. GAPDH was used as the loading control. Relative mRNA expression levels were assessed using the 2−*ΔΔ*CT method, as previously described [13]. Gene-specific primers of MMPs were designed according to published articles [14]. The used primers are listed in Table 1.

### 2.6. Western Blot Analysis

The effect of apatinib on the expression of MMP and TIMP proteins was determined by Western blot analysis, using the same experimental grouping as for the real-time PCR assay. The cells were lysed to extract the total protein, and the protein concentration was determined by the BCA kit according to its instructions. Each protein sample (20 *μ*g) was loaded, and SDS-PAGE was performed on a 12% separation gel. Afterwards, the proteins were transferred from the gel to a PVDF membrane and treated with the respective antibodies: mouse anti-human MMP-3, MMP-9, and MMP-10; mouse anti-human TIMP-3 (1 : 200); rabbit anti-human MMP-1 (1 : 200); and rabbit anti-human MMP-2, MMP-7, MMP-10, MMP-16, TIMP-1, TIMP-2, and TIMP-4 (1 : 500) at 4°C overnight. GAPDH was used as the loading control. The next day, the membrane was incubated with the corresponding secondary antibodies labeled with horseradish peroxidase (purchased from Wuhan Google Biotechnology Ltd., Wuhan, China) and visualized by a chemiluminescent immunoassay.

## 3. Results

### 3.1. Apatinib Inhibits the Migration of HepG2, Hep3B, Huh7, and SMMC-7721 Cells

**Figure 1:**
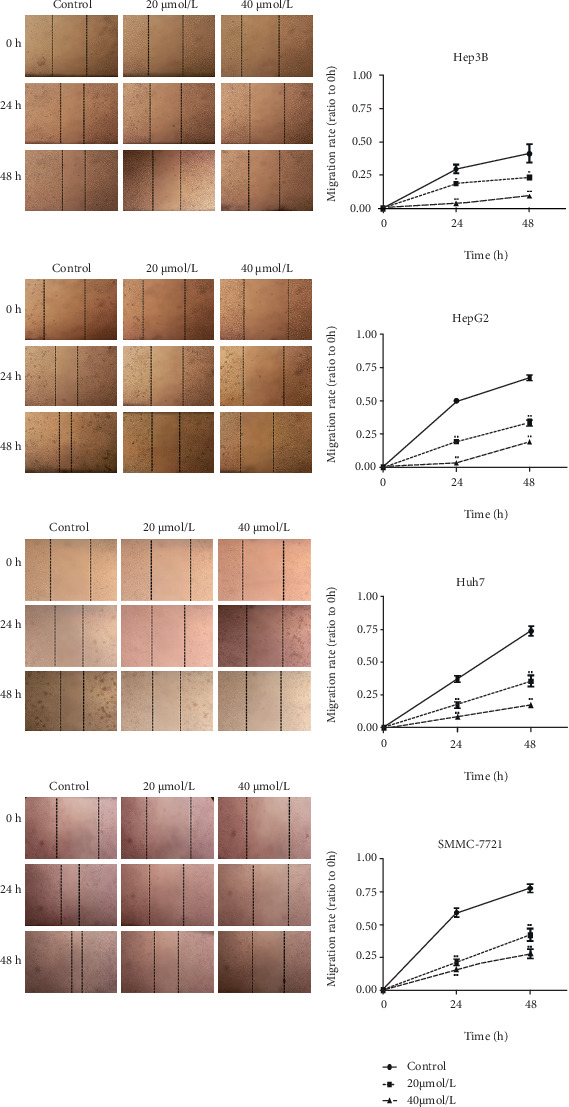
Apatinib inhibits the migration of HepG2, Hep3B, Huh7, and SMMC-7721 cells. Results of the wound healing assay of Hep3B, HepG2, Huh7, and SMMC-7721 cells following treatment with different doses of apatinib at 24 and 48 h (original magnification, ×200). Analysis of the relative migration rate curves showed that apatinib treatment inhibited the migration of Hep3B, HepG2, Huh7, and SMMC-7721 cells in a dose-dependent manner (∗ indicates *p* < 0.05 and ^∗∗^*p* < 0.01 vs. 0 *μ*mol/L (control)).

Wound gaps in the HepG2, Hep3B, Huh7, and SMMC-7721 cell monolayers were observed under a microscope at 0, 24, and 48 h after apatinib treatment. The liver cancer cell lines were incubated with apatinib (20 and 40 *μ*mol/L, respectively) for 48 h, and the results are shown in Figure 1. In contrast to cells in the control group, cells with 24 and 48 h of apatinib treatment obviously migrated into the wound gaps, leading to a significantly reduced migration distance compared with that in the control group (*P* < 0.05). In addition, the migration rate of cells treated with a high concentration (40 *μ*mol/L) of apatinib was significantly lower than that of cells treated with a low apatinib concentration (20 *μ*mol/L), suggesting that apatinib has a significant and dose-dependent inhibitory effect on the migration of liver cancer cells.

### 3.2. Apatinib Inhibits the Invasion of HepG2, Hep3B, Huh7, and SMMC-7721 Cells

**Figure 2:**
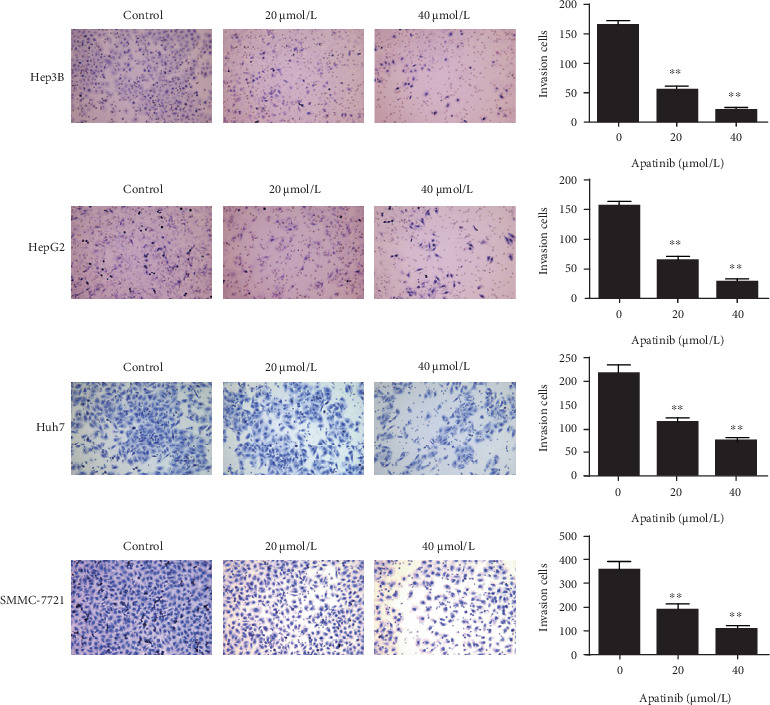
Apatinib inhibits the invasion of HepG2, Hep3B, Huh7, and SMMC-7721 cells. Results of transwell invasion assays of Hep3B, HepG2, Huh7, and SMMC-7721 cells following treatment with apatinib for 24 h (original magnification, ×200). Quantification of the invasion in Hep3B, HepG2, Huh7, and SMMC-7721 cells (∗∗ indicates *p* < 0.01 vs. 0 *μ*mol/L (control)).

The transwell invasion experiment showed that after apatinib treatment, fewer HepG2, Hep3B, Huh7, and SMMC-7721 cells migrated across the filter membrane (Figure 2; *P* < 0.05). In addition, the number of invading cells treated with a high concentration of apatinib was significantly less than that of invading cells treated with a low concentration of apatinib. The results of the four liver cancer cell lines are consistent and indicate that apatinib inhibits the invasion of human liver cancer cells.

### 3.3. Apatinib Downregulates MMP Expression Levels in HepG2 and Hep3B Cells

**Figure 3:**
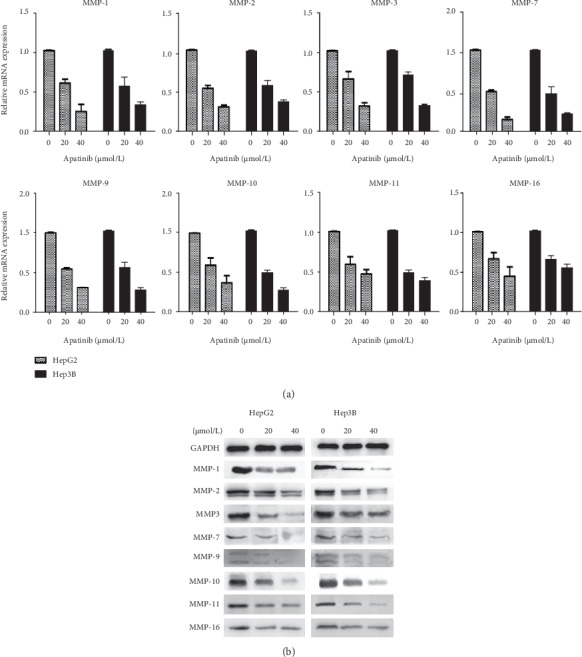
Real-time PCR and Western blot analysis of expression levels of MMP family genes in Hep3B and HepG2 liver cells. (a) Real-time PCR analysis of mRNA levels of MMP-1, MMP-2, MMP-3, MMP-7, MMP-9, MMP-10, MMP-11, and MMP-16. Compared with the control group, the expression levels of the mRNAs of MMP-1, MMP-2, MMP-3, MMP-7, MMP-9, MMP-10, MMP-11, and MMP-16 in the apatinib-treated group were significantly decreased (*p* < 0.05). (b) Western blot was used to screen the level of MMP expression in the HCC cell lines, showing that apatinib reduced the expression of MMP-1, MMP-2, MMP-3, MMP-7, MMP-9, MMP-10, MMP-11, and MMP-16. GAPDH was used as the loading control. MMP: matrix metalloproteinase.

Real-time PCR showed that apatinib treatment obviously reduced the expression levels of MMP-1, MMP-2, MMP-3, MMP-7, MMP-9, MMP-10, MMP-11, and MMP-16 in HepG2 and Hep3B cells compared with the corresponding levels in the control group, and these reductions were statistically significant (*P* < 0.05; Figure 3(a)). Western blot analysis showed that apatinib also downregulated the expression of the abovementioned MMPs at the protein level (Figure 3(b)).

### 3.4. Downregulation of MMPs by Apatinib Is Associated with Upregulation of TIMP3/4 Expression

**Figure 4:**
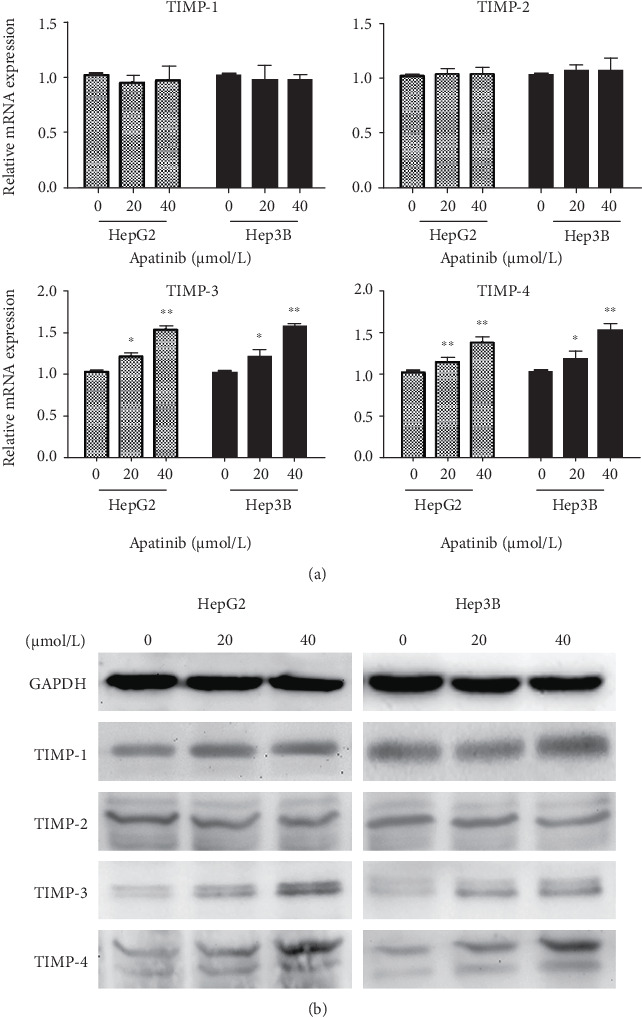
Real-time PCR and Western blot analysis of expression levels of TIMP family genes in Hep3B and HepG2 liver cells. (a) Real-time PCR analysis of the expression levels of TIMP-1, TIMP-2, TIMP-3, and TIMP-4. The results show that apatinib increased the expression of TIMP-3 and TIMP-4. (b) Protein expression levels of TIMPs following treatment with apatinib in Hep3B and HepG2 cells analyzed by Western blot, confirming an increased TIMP-3 and TIMP-4 expression upon apatinib treatment. GAPDH was used as the loading control. TIMP: tissue inhibitors of metalloproteinase.

We further investigated the mechanisms underlying the inhibitory effect of apatinib on HepG2 and Hep3B cell metastasis. The expression levels of members of the TIMP gene family, including TIMP-1, TIMP-2, TIMP-3, and TIMP-4, were analyzed using real-time PCR and Western blot. As shown in Figures 4(a) and 4(b), apatinib treatment had no effect on the expression of TIMP-1 and TIMP-2 in HepG2 or Hep3B cells, although the mRNA and protein levels of TIMP-3 and TIMP-4 were markedly increased. These results indicate that apatinib treatment upregulated the expression levels of members of the TIMP gene family.

### 3.5. Apatinib Downregulates the Activation of the NF-*κ*B Signaling Pathway

**Figure 5:**
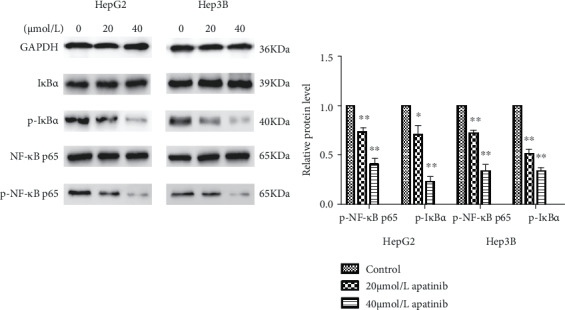
Western blot analysis of expression levels of the NF-*κ*B signaling pathway in Hep3B and HepG2 liver cells. The protein expressions of p65, p-p65, I*κ*B*α*, and p-I*κ*B*α* in Hep3B and HepG2 liver cells measured by Western blotting. Results revealed that the expression levels of p-I*κ*B*α* and p-p65 were decreased in HepG2 and Hep3B liver cancer cells treated with apatinib when compared with the control group in a dose-dependent manner. And the ratio of p-I*κ*B*α*/I*κ*B*α* and p-p65/p65 in cells was significantly lower compared with that of the control group. ImageJ software was used to analyze the gray values. ∗ indicates *p* < 0.05, ^∗∗^*p* < 0.01.

The NF-*κ*B signaling pathway plays an important role in cancer migration [15]; therefore, we explored whether apatinib could affect this pathway. As shown in Figure 5, Western blot analysis revealed that the expression levels of p-I*κ*B*α* and p-p65 were decreased in a dose-dependent manner in liver cancer cells treated with apatinib when compared with the levels in the control group. In addition, the ratios of p-I*κ*B*α*/I*κ*B*α* and p-p65/p65 in HepG2 and Hep3B cells were significantly lower than those in the control group. These results indicated that apatinib inhibits NF-*κ*B signaling pathway activation.

## 4. Discussion

More than half of liver cancer patients are diagnosed after progressing to the advanced stages of liver cancer where surgery is impossible. Many liver cancer patients who do undergo operation also experience local recurrences and distal metastases [16, 17]. MMP proteins degrade extracellular matrix (ECM) proteins and therefore play a key role in the invasion and metastasis of tumor cells, including liver cancer cells [18].

In this study, we confirmed the effect of apatinib on the metastasis and invasion of HepG2, Hep3B, Huh7, and SMMC-7721 cells through a wound-healing assay and transwell invasion assay, respectively. Our results showed that apatinib has a significant and dose-dependent inhibitory effect on the metastasis and invasion of these four liver cancer cells. A recently published study by Li et al. also confirmed the anti-invasion and metastasis effects of apatinib on multiple liver cancer cell lines (Hep3B, BEL-7402, HepG2, Huh7, and HCCC-9810) [19]. To further explore the underlying mechanism, we performed real-time PCR and Western blot analysis and verified that apatinib downregulates the expression of MMPs. Therefore, we believe that apatinib probably inhibits the metastasis and invasion of liver cancer cells by downregulating the expression of MMPs. Previous studies have found that apatinib plays a role in the downregulation of MMP-2 and MMP-9 in gastric cancer [20, 21]. However, our study was the first to verify apatinib-induced downregulation of members of the MMP gene family in liver cancer cells. Tumor angiogenesis includes tube formation and proliferation of endothelial cells. Tube formation is mainly mediated by MMPs, whereas proliferation primarily involves VEGF family members; for example, VEGF-induced angiogenesis is mainly mediated by VEGF-2 [22–24]. As a small molecule inhibitor of VEGFR-2, apatinib inhibits tumor angiogenesis in the two ways mentioned above, explaining its good antitumor efficacy. Currently, it is believed that VEGF promotes the metastasis of tumor cells synergistically with MMPs; this phenomenon has been demonstrated in multiple tumors [25–27]. These data suggest that apatinib can also inhibit the invasion and metastasis of liver cancer by inhibiting the VEGF/VEGFR-2 signaling pathway.

Tissue inhibitors of metalloproteinases (TIMPs) are natural inhibitors of MMPs and play an important role in regulating the activation of MMPs [22]. The balance between these two gene families is critical to the invasion and metastasis of tumor cells [28]. Despite evidence of its antitumor effects, whether apatinib can affect the expression of the TIMP gene family has not yet been reported. Our study showed that apatinib regulates the expression of TIMP-3 and TIMP-4. Therefore, we demonstrated for the first time that apatinib upregulates TIMP family members in liver cancer. It has been reported that the expression of TIMPs significantly inhibits the expression of all active MMP family members [29]. These results suggest that the decrease in MMP gene expression levels in response to apatinib administration may be due to the upregulation of TIMP-3 and TIMP-4. Moreover, studies have found that TIMPs are involved in inhibiting cell migration, invasion, and angiogenesis, which is independent of their inhibitory activity on MMPs [30]. Our study indicates that apatinib reduced the invasive and metastatic capabilities of liver cancer cells, at least in part, by downregulating MMP expression and simultaneously upregulating TIMP expression.

Our study involved eight members of the MMP family—MMP-1, MMP-2, MMP-3, MMP-7, MMP-9, MMP-10, MMP-11, and MMP-16—and the expression of all these MMPs was significantly downregulated by apatinib. Previous research has shown enhanced levels of various MMPs in several types of tumors, including gastric cancer and liver cancer. Łukaszewicz-Zając et al. also demonstrated that high MMP expression in the liver is related to a poorer prognosis of liver cancer patients [31]. MMPs have been indicated to mainly affect the invasion and metastasis of cancer, but studies have also found that some MMPs participate in multiple stages of carcinogenesis [32, 33], such as the destruction of the host immune response, promotion of early tumor progression, and inhibition of cancer cell apoptosis and death. Among all MMPs, MMP-1, MMP-2, MMP-3, MMP-7, and MMP-9 are involved in almost all stages of tumor growth, angiogenesis, and metastasis [34]. Therefore, in addition to inhibiting VEGFR-2, apatinib can further enhance its antitumor effects by downregulating the expression of MMPs.

Studies have reported that upregulation of the nuclear factor-kappaB (NF-*κ*B) signaling pathway can cause the invasion and metastasis of human cancer [35, 36]. Further studies confirmed that inhibiting NF-*κ*B signaling pathway activity significantly reduced invasion of HCC cells and downregulated the expression of the VEGF and MMP gene families, the latter including MMP-1, MMP-2, MMP-3, and MMP-9 [37, 38]. These observations indicate that apatinib inhibits the migration and invasion of liver cancer cells by decreasing MMP-related gene expression via suppression of the NF-*κ*B signaling pathway.

In conclusion, apatinib inhibits the invasion and metastasis of liver cancer via regulation of the MMP/TIMP balance. Therefore, apatinib is a potential antimetastasis drug for liver cancer. Next, we will validate this conclusion in animal models and further explore the antimetastasis mechanism of apatinib to provide a basis for a better application of apatinib in the clinic.

## Figures and Tables

**Table 1:** Characteristics of the primers used for real-time PCR.

Genes	Primers (forward and reverse)	Products (bp)
MMP-1	5′-CTGCTTACGAATTTGCCGACAGA-3′	130
	5′-GTTCTAGGGAAGCCAAAGGAGCTG-3′	
MMP-2	5′-GTTCATTTGGCGGACTGTGACG-3′	146
	5′-ATTCATTCCCTGCAAAGAACACAGC-3′	
MMP-3	5′-GGACAAAGGATACAACAGGGACCA-3′	140
	5′-GAACCGAGTCAGGTCTGTGAGTG-3′	
MMP-7	5′-GATGGGCCAGGAAACACGC-3′	107
	5′-CCTAGACTGCTACCATCCGTCCA-3′	
MMP-9	5′-CACGACGTCTTCCAGTACCGAGA-3′	115
	5′-CATAGGTCACGTAGCCCACTTGGT-3′	
MMP-10	5′-TCTGTTCCTTCGGGATCTGAGATG-3′	146
	5′-TGAAATTCAGGTTCAGGGTTCCAGT-3′	
MMP-11	5′-ACCTGGACTATCGGGGATGA-3′	188
	5′-GGCTGGCCATATAGGTGTTG-3′	
MMP-16	5′-GACAGGCCAAAACCTCCTCGG-3′	149
	5′-TTTCTCACTCGCCAAAACCACTG-3′	
TIMP-1	5′-AAGGCTCTGAAAAGGGCTTC-3′	161
	5′-GAAAGATGGGAGTGGGAACA-3′	
TIMP-2	5′-TTGACCCAGAGTGGAACG-3′	101
	5′-ACCAAAGACGGGAGACGA-3′	
TIMP-3	5′-CCTGCTACTACCTGCCTTGC-3′	182
	5′-TGTGGCATTGATGATGCTTT-3′	
TIMP-4	5′-TCTGGACAGACTGGCTGTTG-3′	176
	5′-TTGAAGGGATGTGATGGTCA-3′	
NF-*κ*B p65	5′-CAAGTGGCCATTGTGTTCCG-3′	149
	5′-TGGCGATCATCTGTGTCTGG-3′	
GAPDH	5′-TCGACAGTCAGCCGCATCTTCTTT-3′	148
	5′-GCCCAATACGACCAAATCCGTTGA-3′	

MMP: matrix metalloproteinase; TIMP: tissue inhibitors of metalloproteinase.

## Data Availability

The data used to support the findings of this study are available from the corresponding authors upon request.
